# Probing the luminal microenvironment of reconstituted epithelial microtissues

**DOI:** 10.1038/srep33148

**Published:** 2016-09-13

**Authors:** Alec E. Cerchiari, Karen E. Samy, Michael E. Todhunter, Erica Schlesinger, Jeff Henise, Christopher Rieken, Zev J. Gartner, Tejal A. Desai

**Affiliations:** 1 UC Berkeley – UCSF Graduate Program in Bioengineering, Berkeley - Berkeley (CA), USA.; 2 Department of Bioengineering and Therapeutic Sciences, UCSF, San Francisco (CA), USA; 3 Department of Pharmaceutical Chemistry, UCSF, San Francisco (CA), USA; 4 Prolynx LLC, San Francisco (CA), USA.; 5 Carl Zeiss Microscopy, LLC, Thornwood (NY), USA.

## Abstract

Polymeric microparticles can serve as carriers or sensors to instruct or characterize tissue biology. However, incorporating microparticles into tissues for *in vitro* assays remains a challenge. We exploit three-dimensional cell-patterning technologies and directed epithelial self-organization to deliver microparticles to the lumen of reconstituted human intestinal microtissues. We also develop a novel pH-sensitive microsensor that can measure the luminal pH of reconstituted epithelial microtissues. These studies offer a novel approach for investigating luminal microenvironments and drug-delivery across epithelial barriers.

Three-dimensional (3D) culture systems allow epithelial cells to self-organize into structures comparable to their *in vivo* architecture. Emerging 3D culture models facilitate drug testing, tissue engineering, and the study of tissue morphogenesis in a biologically relevant context. Under these biomimetic 3D culture conditions, epithelial cells can proliferate, organize, and polarize into lumenized multicellular structures frequently referred to as cysts. In a cyst, epithelial cells tend to orient their apical surfaces towards the lumen and their basal surfaces towards the extracellular matrix (ECM) where integrins and other receptors are bound and engaged. Epithelial cells also interact with neighboring cells through specialized lateral junctions such as adherens junctions, tight junctions, or desmosomes^1^. In conjunction with cell-ECM adhesion complexes such as focal adhesions or hemi-desmosomes, cell-cell and cell-ECM interactions coordinate the assembly of a continuous monolayer surrounding a luminal space.

The human colon adenocarcinoma cell line, Caco-2, efficiently forms lumenized cysts in 3D culture. Caco-2 cells are commonly used in the drug discovery process to predict transepithelial permeability and in the basic sciences to study the fundamentals of tissue morphogenesis^2^,^3^. In the context of drug discovery, Caco-2 monolayers are typically cultured in two-dimensional (2D) transwell systems. In this format, Caco-2 cells can develop a polarized epithelial barrier that shares many of the properties of small intestine columnar epithelia^4^. Furthermore, transport studies have shown that permeability across these confluent monolayers correlates to human intestinal absorption for high permeability drugs^5^,^6^. Thus, the Caco-2 monolayer model has proven useful not only for mechanistic studies of drug absorption but also as an absorption-screening assay for preclinical drug selection.

Nevertheless, the 2D nature of the transwell system neglects the effects of a 3D luminal microenvironment such as compartmentalized pH levels, metabolite concentration gradients, and the various implications of curved, three-dimensional geometry^7^. Therefore, studying the epithelial barrier of 3D Caco-2 cysts could reveal new insights about how therapeutics cross intestinal barriers *in vivo*. However, the only method for delivering cargo molecules to a 3D lumen is microinjection – a technically complicated method that is intrusive (i.e. must perforate the epithelial barrier), unsuitable for physically large cargo, and inherently low-throughput^8^,^9^. We therefore sought an approach to non-invasively introduce microparticles incorporating drugs or sensors within the lumen of Caco-2 cysts.

## Results

### Polymeric microparticles can be accommodated within the lumen of Caco-2 cysts

**Figure 1:**
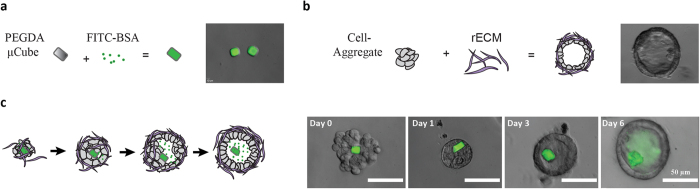
Non-intrusive delivery of microparticles to the luminal compartment of a reconstituted cyst. (**a**) Schematic illustration and 40X image of cuboidal (15 μm × 15 μm × 15 μm) microparticles made from polyethylene glycol diacrylate (PEGDA) and loaded with FITC-BSA. (**b**) Schematic illustration and 40X phase contrast image of lumenized cyst reconstituted via Sacrificial Micromolding of a Caco-2 cell-aggregate in Matrigel – a reconstituted extracellular matrix (rECM). (**c**) Schematic illustration (left) and 40X images (right) of how Sacrificial Micromolding into Matrigel can be used to incorporate the cuboidal PEGDA microparticles into the core of Caco-2 cell-aggregates capable of undergoing morphogenesis, while also retaining the microparticle within the luminal compartment of the cyst.

To deliver a microparticle to the interior of a multicellular cyst, we sought a strategy that would allow small collections of cells to grow and develop around a polymeric microparticle. Based on our analytical model for tissue self-organization^10^, we reasoned that the higher affinity of epithelial cells for adhesive ECM relative to a non-adhesive microparticle could direct an aggregate of cells to push a microparticle into their luminal space when fully embedded in adhesive ECM. We previously demonstrated a technique called Sacrifical Micromolding that allows collections of 10 to 50 epithelial cells to be aggregated, fully embedded in biomimetic hydrogels such as Matrigel or collagen, and self-organize into a lumenized cyst^11^. We therefore used Sacrificial Micromolding^10^ to pre-aggregate Caco-2 cells and polymeric microparticles within degradable microwells, before transferring these aggregates to Matrigel culture. We initially explored cuboidal microparticles (15 μm × 15 μm × 15 μm) fabricated from polyethylene glycol (PEG) and photolithographically shaped as previously described^12^. Using Sacrificial Micromolding, we found that approximately 25% of the multicellular aggregates that hosted one microcube developed into cysts incorporating that cube within their lumen, as shown in Fig. 1. Using an alternative approach that relied on passive pre-aggregation in non-adhesive microwells and subsequent transfer to Matrigel^10^, we found that only 2–3% of cysts incorporated the cube within their lumen. Nonetheless, due to *i)* the high-volume of multicellular aggregates transferred from agarose to Matrigel in each experiment (at least 3000 to 5000), *ii)* the ease of identifying which aggregates contained microparticles, and *iii)* the simplicity of the approach, we focused on this latter method for further experimentation.

### Microparticle geometry affects actin belt formation and lumen incorporation

**Figure 2:**
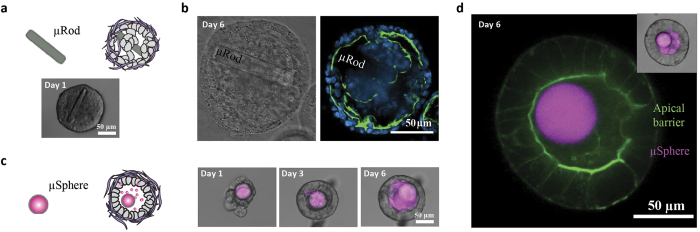
Microparticle geometry affects actin belt formation and lumen incorporation. (**a**) Schematic illustration and 40X phase contrast image showing how microrods (15 μm × 15 μm × 100 μm) can be incorporated within the core of Caco-2 microtissues. (**b**) Representative 40X phase contrast image (left) and confocal slice (right) showing how, after one week in culture, microrods alter luminal clearing and the establishment of the characteristic actin belt lining the luminal compartment of a polarized Caco-2 cyst. (**c**) Schematic illustration (left) and 40X images (right) of PEG microspheres (30 μm in diameter) loaded with FITC-BSA (magenta) and incorporated in lumen. (**d**) 40X confocal slice with widefield inset (top right) of a Caco-2 cyst showing a continuous actin staining signal (green) and a FITC-BSA loaded PEG microsphere (magenta) in the lumen.

During our initial experiments using PEG microcubes, we noticed that slight differences in the size and shape of the cuboidal microparticles appeared to affect lumen formation of reconstituted epithelial cells. We therefore investigated the effects of microparticle geometry on Caco-2 lumen formation. PEG microrods with dimensions of approximately 15 μm × 15 μm × 100 μm were found too large to be incorporated within multicellular aggregates without affecting lumenogenesis. Even though some microrods could be accommodated within the core of the tissues (Fig. 2a), immunofluorescent analysis of actin, a marker of apical polarity, revealed a significant defect in the capacity of these tissues to establish the characteristic actin belt surrounding the lumen of a well-polarized Caco-2 cyst (Fig. 2b). On the other hand, we found that PEG hydrogel microspheres of approximately 30 μm diameter, originally developed as a carrier for drug delivery^13^, were more efficient in localizing to the lumen than their cuboidal counterparts (Fig. 2c,d), despite their larger volume and surface area. Taken together, these data suggest that differences in size and shape of aggregate-embedded microparticles can have profound effects on lumen formation.

### Microparticle composition can trigger tissue inversion or allow nanoparticle delivery

We next sought to evaluate the effects of microparticle composition on the capacity of epithelial cells to lumenize. We pre-aggregated Caco-2 cells with either polystyrene (PS) or maltodextrin microparticles of comparable size and shape. We chose PS because this type of polymer is readily available and routinely used for 3D culture. We chose maltodextrin due to its routine use as an FDA-approved food additive^14^, the simple preparation of maltodextrin microparticles using emulsions^15^, and its capacity to be degraded by secreted intestinal enzymes such as amylase^16^.

**Figure 3:**
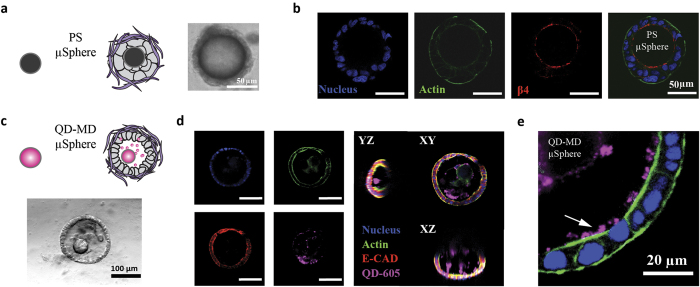
Microparticle physicochemical properties affect tissue polarity. (**a**) Schematic illustration (left) and 40X phase contrast image (right) showing polystyrene (PS) microspheres incorporated into the core of Caco-2 microtissues lacking lumen after one week in 3D Matrigel culture. (**b**) 40X confocal slices through a PS microsphere embedded within a Caco-2 cell aggregate and cultured in Matrigel for one week. Caco-2 cells form a coherent microtissue around the microsphere, but the tissue exhibits inverted polarity with actin (green) preferentially oriented towards the surrounding ECM (i.e. Matrigel) and with β4 integrin (red) localized at the interface between the tissue and the PS-microsphere. Nuclei are stained with DAPI (blue). (**c**) Schematic illustration (top) and 20X phase contrast image (bottom) of a quantum-dot-loaded maltodextrin (QD-MD) microsphere incorporated into the luminal compartment of Caco-2 cysts after one week in culture. (**d**) 40X confocal slices (left) and 20X confocal orthogonal views (right) of a Caco-2 cyst with a QD-MD microsphere within its luminal compartment. (**e**) High magnification 63X confocal slice showing quantum dots trapped within the lumen of the cyst (white arrow).

Unlike PEG microparticles, PS microparticles dramatically perturbed self-organization and cyst formation as shown in Fig. 3a. Although the Caco-2 cells formed coherent microtissues around the microparticles, the basal and apical polarity of these microtissues was completely inverted. β4 integrin was proximal to the internalized PS microparticle and not in contact with the reconstituted ECM (i.e. Matrigel), while actin appeared to be oriented away from the microparticle itself (Fig. 3b). In contrast, bare maltodextrin microparticles and maltodextrin microparticles bearing adsorbed quantum-dots (QD 605) were readily incorporated into Caco-2 cysts and did not obviously perturb self-organization (Fig. 3c). As shown in Fig. 3d,e, we also found that, after one week in 3D culture, quantum dots had dissociated from the microparticle yet remained completely entrapped within the luminal space of the Caco-2 cysts, suggesting that the 3D monolayer prevented escape from the luminal compartment. Taken together, these data illustrate delivery of nanoparticle-laden microparticles to the luminal compartment of epithelial cysts and how the physicochemical properties of the microparticles may trigger undesirable phenomena such as tissue inversion.

### Polymeric microsensors permit the study of reconstituted luminal microenvironments

**Figure 4:**
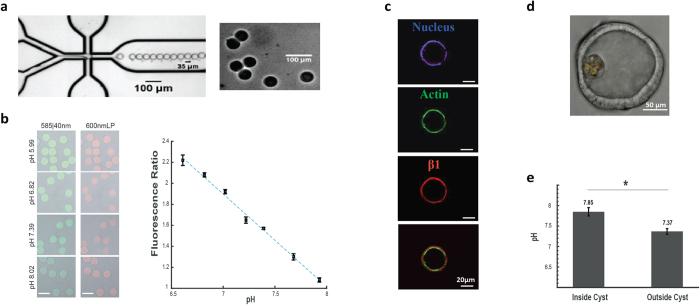
Probing the luminal pH of epithelial tissues. (**a**) Sample images of the microfluidic device (left) used to produce 35 μm diameter PEG microparticles that swell to an average diameter of 45 μm in physiological buffers (right). (**b**) Confocal slices showing SNARF-conjugated PEG microparticles with pH-dependent fluorescent profiles (left) and corresponding calibration curve quantifying the ratiometric fluorescent intensity of the microparticles as a function of pH (right). Scale bars are 50 μm. (**c**) 10X confocal slice of a Caco-2 cyst exhibiting correct basal and apical polarization upon microparticle incorporation. (**d**) Sample 20X image of a Caco-2 cyst incorporating a SNARF-conjugated PEG microparticle within the luminal compartment and (**e**) quantification of luminal pH within cysts as opposed to surrounding pH outside cysts (n = 5) as determined by ratiometric fluorescent intensities of SNARF-conjugated PEG microparticles.

Following the successful delivery of nanoparticles to the luminal space of Caco-2 cysts using degradable microparticles, we sought to further validate our approach by delivering sensors to these lumen. Specifically, we aimed to deliver microsensors capable of characterizing the luminal pH of Caco-2 cysts with seminaphtharhodafluor (SNARF) - a fluorescent dye used as a ratiometric pH indicator^17^,^18^. We conjugated SNARF to microparticles constructed from tetra-PEG hydrogel using an adaptation of previously published methods^19^ (Fig. 4a). Calibration curves for microparticle-conjugated SNARF concentrations between 0.02 and 0.03 mM allowed for ratiometric fluorescent measurements from pH 6.61 to 8.02 in either microparticle or bulk-gel form (Fig. 4b *and* Supplementary Information). Upon delivering the pH-sensitive microparticle to the luminal compartment of reconstituted epithelial cysts, immunofluorescent staining of actin and β1 integrins indicated correct polarization of the Caco-2 cysts, suggesting that the non-adhesive PEG-SNARF microparticles did not affect the self-organization or polarization of these cells (Fig. 4c,d). Using the microparticles, we also found that, after one week in culture, the lumen of Caco-2 cysts exhibited a more alkaline pH than the surrounding medium (Fig. 4e *and* Supplementary Information; p < 0.05 one-way ANOVA). Taken together, these data demonstrate a new approach for luminal delivery of sensors capable of probing the luminal microenvironment of epithelial microtissues.

## Discussion

Polymeric microparticles such as those used in this study are gaining interest as both drug carriers and sensors. Polymeric sensors can sense specific biological signals, such as release of proteins or antibodies in response to tissue damage and inflammatory events, detect small molecules like glucose and lactose, and monitor pH in real-time and at low concentrations. Despite the advantages of these polymeric sensors to sense biological signals in cellular microenvironments, progress has been limited due to the challenges associated with incorporating these structures into tissues. To the best of our knowledge, this is the first report describing the incorporation of polymeric microparticles into the luminal compartment of a reconstituted epithelial cyst without perturbing tissue self-organization^20^. Using Caco-2 microtissues, we found that small, spherical, and fairly non-fouling polymeric microparticles did not appear to have profound effects on epithelial lumenogenesis. Interestingly, our experiments using PS microparticles did perturb self-organization, yielding microtissues with inverted polarity. It is possible that the rigid and hydrophobic nature of PS may promote the adsorption of secreted ECM molecules and trigger the engagement of cell-ECM adhesion complexes that drive the establishment of this inverted polarity^21^,^22^. In contrast, minimally adhesive polymers such as PEG or maltodextrin were better choices for our intended studies. In particular, the rich history of PEG as a functionalizable and degradable polymer that can act as a carrier of sensors or therapeutics^23^,^24^,^25^ makes this material an exquisite choice for future work. In this proof-of-concept, we use functionalized tetra-PEG hydrogel microparticles to probe the pH of 3D Caco-2 lumen, but we also foresee using degradable PEG microconstructs of increasing complexity in order to non-intrusively deliver therapeutics and other types of nanosensors. For example, there is potential to program tunable and predictable pH-dependent hydrogel degradation and drug-release profiles into PEG microparticles by incorporating cleavable linkers as crosslinks and tethers for drug payloads^13^,^26^. While the biological events that allow the internalization of these polymeric microstructures into the lumen of the epithelial cysts require more detailed investigation, our previous reports suggest that the preferential affinity of epithelial cells for ECM over non-fouling surfaces directs the passive transport of the microparticles away from the ECM interface and towards the cyst lumen. A more in-depth understanding of this process will be key to the design and development of efficient drug carrier systems and sensors for various applications. However, this study provides a proof-of-concept mechanism of payload delivery into the lumen of Caco-2 cysts. This paves the way for the possibility of continuous real-time monitoring of analytes within microtissues of increasing complexity, meeting an important need for the field.

## Methods

### Photolithographic techniques: Micromolding and Rectangular Microparticles

Freestanding SU-8 features were fabricated on silicon wafers using standard photolithographic techniques at the Biomedical Micro- and Nano-Fabrication Center (BMNC) at UCSF. For Sacrificial Micromolding in Matrigel, a detailed description of the adapted photolithographic methods used can be found in ref. 11. For multicellular pre-aggregation in non-adhesive agarose wells and transfer to Matrigel, a detailed description of the methods used can be found in ref. 10. For a detailed description of how cuboidal or rod-like PEG-microparticles were fabricated via photolithography, see refs 12 and 27, respectively.

### Polystyrene Microsparticles, Maltodextrin Microparticles, and Quantum Dot Physisorption

The 75 μm diameter polystyrene beads were purchased from Polysciences Inc (Catalog # 24049-5). For maltodextrin microparticles. 2 g of 4–7 dextrose-equivalent maltodextrin was dissolved in 2 M NaOH. 2 mL of this solution added to 18 mL of 4:1 (v/v) cyclohexane:chloroform with 1% (v/v) Span-80 in a SigmaCote-silanized scintillation vial. This mixture was emulsified by vortexing for 20 seconds, then 400 μL of epichlorohydrin was immediately stirred into the emulsion. The reaction proceeded for 1 hr at 40 °C with spinning at 1200 rpm. The emulsion was then washed twice with cyclohexane and four times with distilled water. The resultant polymerized maltodextrin was run through a 100 μm mesh and then a 40 μm mesh to filter out large particles. The beads were assayed for amylase degradability and then used as described. The beads were stored in distilled water at room temperature until use. Quantum dots were loaded onto maltodextrin microparticles by adsorption. Quantum dots (Qdot 605 ITC carboxyl quantum dots, Thermo Fisher) were mixed with 5% of the microparticle prep (equivalent to 100 mg maltodextrin) and incubated overnight in the dark at 4 °C in 1 mL of PBS. Microparticles were separated from free quantum dots by centrifugation at 200 g.

### SNARF-derivatized Tetra-PEG Hydrogel Microparticles

SNARF-derivatived microparticles were based on biodegradable tetra-PEG hydrogel microparticles originally developed for controlled drug delivery as described in ref. 19. These gels self-assemble upon mixing two solutions containing functionalized four-armed PEG prepolymers (Prepolymer A and Prepolymer B), through a strain-promoted azide–alkyne cycloaddition (SPAAC) crosslinking reaction. This reaction, also referred to as copper-free click chemistry (see ref. 28), occurs between four azide (Prepolymer A) and four cyclooctyne (Prepolymer B) end groups to form stable triazole crosslinks between the two tetra-PEG prepolymers. In addition to the azido end-groups used for crosslinking, Prepolymer A also contains free amine end-groups for conjugation to a payload. Here, we attached SNARF to those amino groups, using standard amide-bond-forming chemistry, by acylation with SNARF-NHS ester (Invitrogen S22801) to give a SNARF-functionalized Prepolymer A. Microparticles were formed upon mixing with a solution of the cyclooctyne-derivitized Prepolymer B in a flow-focusing microfluidic drop-forming device (Fig. 4a). For a detailed synthetic procedure for the SNARF-derivatized prepolymers, see Supplementary Information.

### Cell Culture and Microparticle-cell Mixtures

Caco-2 cells were maintained in 2D cell cultures as described in ref. 29. After dissociation from culture plates, Caco-2 cells were resuspended in 0.5 ml of EMEM media at a concentration of 1 M cells/ml. The microparticles were added to the cells at a target concentration of 1 microparticle per 100 cells, and 0.5 ml of the particle-cell mixture was pipetted onto the photolithographically defined micromolds (i.e. agarose or gelatin microwells) before proceeding as described in refs 10 and 11. 3D cell cultures were monitored for lumenization and polarity by phase-contrast, fluorescence, or confocal microscopy as discussed in text. For all experiments, Matrigel (354230; Lot # 07898) was obtained from BD Biosciences.

### Image acquisition

A spinning disk confocal microscope (Zeiss Cell Observer Z1 equipped with a Yokagawa spinning disk and running Zeiss Zen Software) was used to acquire confocal microscopy images, while an inverted epifluorescence microscope (Zeiss Axiovert 200 M running SlideBook software) was used to acquire all other images in this study.

### Immunofluorescence

Microtissues were fixed with 4% formaldehyde in PBS for 20 minutes followed by incubation in blocking buffer (10% heat-inactivated goat serum in PBS + 0.5% Triton X-100) at 4 °C for at least 1 day. Primary antibodies were diluted to 1:50 in blocking buffer and added to the sample. After another incubation period of at least 1 day at 4 °C with the primary antibody, microtissues were washed several times with PBS and incubated with Alexa-conjugated secondary antibodies (1:200 in blocking buffer) for approximately 1 day. All sample were extensively washed with PBS + Triton X-100 + 1 μg/mL DAPI before imaging.

### pH Measurements

SNARF1-functionalized PEG microparticles were suspended in a buffer series from pH 6.61 to 7.93 and imaged on the spinning disk confocal to determine the calibration curve. The ratiometric dye was imaged with a 561 nm laser and fluorescent emission light was collected with a 585/40 nm bandpass filter (f_585_) and a 600 nm longpass filter (f_600_). The calibration curve was prepared by calculating the ratio of (f_585_)/(f_600_) (Fig. 4b). Experimental data was collected using the same settings, and the pH of the lumen was determined using the calibration curve obtained for the microparticles in suspension. For a detailed discussion of the caveats that relate to these measurements and alternative approaches for determining the luminal pH of the cyst see Supplementary Information.

## Additional Information

**How to cite this article**: Cerchiari, A. E. *et al*. Probing the luminal microenvironment of reconstituted epithelial microtissues. *Sci. Rep.* **6**, 33148; doi: 10.1038/srep33148 (2016).

## Supplementary Material

Supplementary Information
