# Supplementary material for: Probing the luminal microenvironment of reconstituted epithelial microtissues
Source: Sci Rep. 2016 Sep 13;6:33148. doi: 10.1038/srep33148 (PMC5020616; doi:10.1038/srep33148)
Supplement: Supplementary Information [file srep33148-s1.doc]

**Probing the luminal microenvironment of reconstituted epithelial microtissues**

Alec E. Cerchiari1,2,3 (‘), Karen E. Samy1,2 (‘), Michael E. Todhunter3, Erica Schlesinger1,2, Jeff Henise4, Christopher Rieken5, Zev J. Gartner1,3, Tejal A. Desai1,2 (*)

1 UC Berkeley – UCSF Graduate Program in Bioengineering, Berkeley - Berkeley (CA); 2 Department of Bioengineering and Therapeutic Sciences, UCSF, San Francisco (CA); 3 Department of Pharmaceutical Chemistry, UCSF, San Francisco (CA); 4 Prolynx LLC, San Francisco (CA); 5 Carl Zeiss Microscopy, LLC, Thornwood (NY);

(‘) *These authors contributed equally*

(*) *Corresponding author*

**Supplementary Information**

*Abbreviations*

MFCO, monofluoro-cyclooctyne, DIPEA, N,N-diisopropylethylamine; PEG20kDa, 4-armed 20,000 MW polyethylene glycol; TNBS, 2,4,6-Trinitrobenzenesulfonic acid; MTBE, methyl tertiary-butyl ether; EtOAc, ethyl acetate; NHS, N-hydroxysuccinimide; THF, tetrahydrofuran; DCC, N,N'-dicyclohexylcarbodiimide; TFA, trifluoroacetic acid; DCM, dichloromethane; DMF, dimethylformamide.

*Synthesis and calibration of SNARF derivatized tetra-PEG hydrogel microspheres.*

*General*

PEG20kDa-(NH2.HCl)4 was purchased from JenKem (item A7026, 4ARM-NH2-20K). DBCO-acid was purchased from Click Chemistry Tools (item 1117). DMF was stored over an AldraAmine packet (Sigma, Item Z511706) to reduce dimethylamine and water contamination. All other commercially available reagents were of reagent grade purity and were used without further purification. Trinitrobenzene sulfonate (TNBS) assays to were conducted to quantify amines on PEG conjugates and the concentration of azides in solution was determined spectrophotometrically by reaction with DBCO-acid as previously described1, however Δe308 = 13,448 M−1·cm−1 was used. Absorption analyses were performed using a Hewlett-Packard 8453 UV-Vis spectrophotometer.

*Materials*

***PEG20kDa-[MFCO]****4 (****Prepolymer B****)*was synthesized according to published procedures2.

.

***6-Azidohexyl-succinimidyl carbonate*** was synthesized and characterized according to a published procedure3.

***N3-Lys(Boc)-OH*** was synthesized according to a published procedure2, with the change that the stable linker 6-azidohexyl-succinimidyl carbonate was used in place of analogous cleavable-crosslinkers used to make biodegradable hydrogels.A solution of H-Lys(Boc)-OH (200 mg, 0.81 mmol, 1.0 equiv) in aqueous NaHCO3 (0.5 M, 8 mL) was treated with a solution of 6-azidohexyl-succinimidyl carbonate (242 mg, 0.85 mmol, 1.05 equiv) in acetonitrile (8 mL). The reaction was allowed to stand for 2 h then assessed for residual amines by TNBS assay (<0.5% amines remained). After concentration to 5 mL under reduced pressure the reaction mixture was acidified to pH 2.5 with 1N HCl then extracted with EtOAc (4 x 10 mL). The combine EtOAc extracts were washed with water (3 x 10 mL), then brine (10 mL), and concentrated to dryness under reduced pressure to provide N3-Lys(Boc)-OH(273 mg,81 % yield)as a clear oil. ESI MS, M+H calc. 416.3, found: 416.2.

***[N3-Lys(Boc)-NH]4-PEG20kDa***was synthesized according to a published procedure2, for analogous prepolymers. Neat N3-Lys(Boc)-OH(70 mg, 0.17 mmol, 1 equiv) was treated with a solution of NHS (20.4 mg, 0.177 mmol, 1.05 equiv) in THF (1.5 mL), and a solution of DCC (36.5 mg, 0.177 mmol, 1.05 equiv) in THF (1.5 mL) at 4 °C. The resulting mixture was allowed to stand for 18 h at 4 °C, then filtered through a cotton plug and added to a solution of PEG20kDa-(NH2.HCl)4 (700 mg, 0.14 mmol (NH2), 0.82 equiv) and DIPEA (36.4 mg, 0.28 mmol, 2 equiv) in acetonitrile (5.5 mL). After 2 h the reaction mixture was assessed for unreacted amines by TNBS assay (<1% amines remained). To cap unreacted amines the reaction was treated with AC2O (17.3 mg, 0.17 mmol, 1 equiv per NH2)for 1 hour. The reaction mixture was concentrated to 2.5 mL then treated with Et2O (50 mL). The resulting precipitate was recovered by filtration, washed with Et2O (4 x 50 mL), and dried under vacuum to give [N3-Lys(Boc)-NH]4-PEG20kDa (650 mg, 93% yield) as a white solid.

***[N3-Lys(H)-NH]4-PEG20kDa****(****Prepolymer A****)*was synthesized according to a published procedure2,for analogous prepolymers. A solution of [N3-Lys(Boc)-NH]4-PEG20kDa(644 mg) in 50% (v/v) TFA in DCM was kept for 1.5 h, then concentrated to dryness under reduced pressure. The residue was dissolved in methanol (20 mL) and the resulting solution was concentrated to 1.5 mL, then treated with Et2O (50 mL). The resulting precipitate was recovered by filtration, washed with Et2O (4 x 50 mL) and dried under vacuum to give [N3-Lys(H)-NH]4-PEG20kDa ­(594 mg, 92% yield).

(Note: SNARF loaded to 5.2% of total capacity)

***[N3-Lys(SNARF)-NH]4-PEG20kDa****(****SNARF-Prepolymer A****)*

A solution of [N3-Lys(H)-NH]4-PEG20kDa (0.797 mL, 20.6 mM, 0.0164 mmol NH2, 1 equiv) in 10 mM pH 5.0 acetate was treated with 0.2 M, pH 8.5 HEPES buffer (0.299 mL) followed by a solution of SNARF-1 NHS ester (Invitrogen S22801, 0.472 mL, 0.18 mM, 0.00085 mmol, 0.052 equiv) in DMF. The resulting mixture was kept for 1 hour then, to cap excess amines, treated with a solution of Ac-NHS (500 mM, 0.066 mL, 0.033 mmol, 2 equiv) in DMF for 30 minutes. This reaction mixture contained 10 mM azide, and 0.05 mM SNARF as the final concentrations, it was filtered through a 0.2 um filter, and used as is in microfluidic devices to make microspheres.

*Microfluidic production of hydrogel microspheres*

SNARF-prepolymer A and prepolymer B both at a concentration corresponding to 10 mM azide or MFCO end-groups were processed into microspheres using microfluidic methods exactly as described for analogous hydrogels (see reference 2).

*SNARF calibration*

Carboxyseminaphthorhodaﬂuor-1 (C-SNARF-1 or SNARF) is a ﬂuorescent ratiometric probe that allows pH quantiﬁcation independent of probe concentration and/or laser intensity. Even though C-SNARF-1 has seen considerable use in studying [H+] in eukaryotic systems4, C-SNARF emission characteristics can be influenced by the probe’s interaction with various cell and matrix components5,6, and these interactions must be controlled for accurate measurements. This has prompted efforts to critically examine the potential use of C-SNARF-1 as a reliable quantitative indicator of pH. One study has investigated the potential of a ﬂuorinated derivative of C-SNARF-1, C-SNARF-4, as a quantitative indicator of pH microenvironments in microbial bioﬁlms by determining the extent to which bioﬁlm matrix components inﬂuence C-SNARF-4 emission properties7. The authors concluded that from pH 5.6 to 7.0, there was little difference in C-SNARF-4 emissions whether in pH-adjusted buffer or in the presence of matrix components. Above pH 7.2, however, bacterial cell/C-SNARF-4 samples revealed a slight increase in ﬂuorescence intensity ratio and showed even greater variation at pH 7.6. However, these increases were attributed to internalization of the pH probe by the bacterial cells, which would influence emission intensity due to the more alkaline intracellular pH. The authors also observed that variations at pH 7.6 translated into a difference of only 0.2 pH units and concluded that C-SNARF-4 can be used as a reliable quantitative indicator of pH in microenvironments of P. aeruginosa bioﬁlms in situ. Other studies have established that lipids as models for cell membranes6,8 as well as double-stranded and heat-denatured DNA8 induce no change in SNARF-1 ﬂuorescence.

These reports have encouraged us to use C-SNARF as a sensor to study the luminal pH of Caco-2 microtissues. However, since no previous studies have examined the spectral emission of SNARF-1 in Matrigel and since Matrigel is known for its high compositional variability, we carried out our own control experiment to further characterize any potential scattering phenomena that may have affected our pH measurements in Figure 4 of the manuscript. First, we obtained a calibration curve using pH-sensitive microparticles fully embedded in Matrigel rather than suspended in solution (Figure S1A). Second, we obtained a calibration curve wherein a monolayer of Caco-2 cells was between the pH-sensitive microparticles and the microscope objective (Figure S1B). Using these calibration curves to calculate the luminal pH reported in Figure 4 adjusts the reported pH from 7.85 to 7.73 or 7.69, respectively for S1A and S1B. These curves appeared substantially similar to each other, suggesting that the upper limit to the optical effects of Matrigel and cells lies within the error of the experiment.


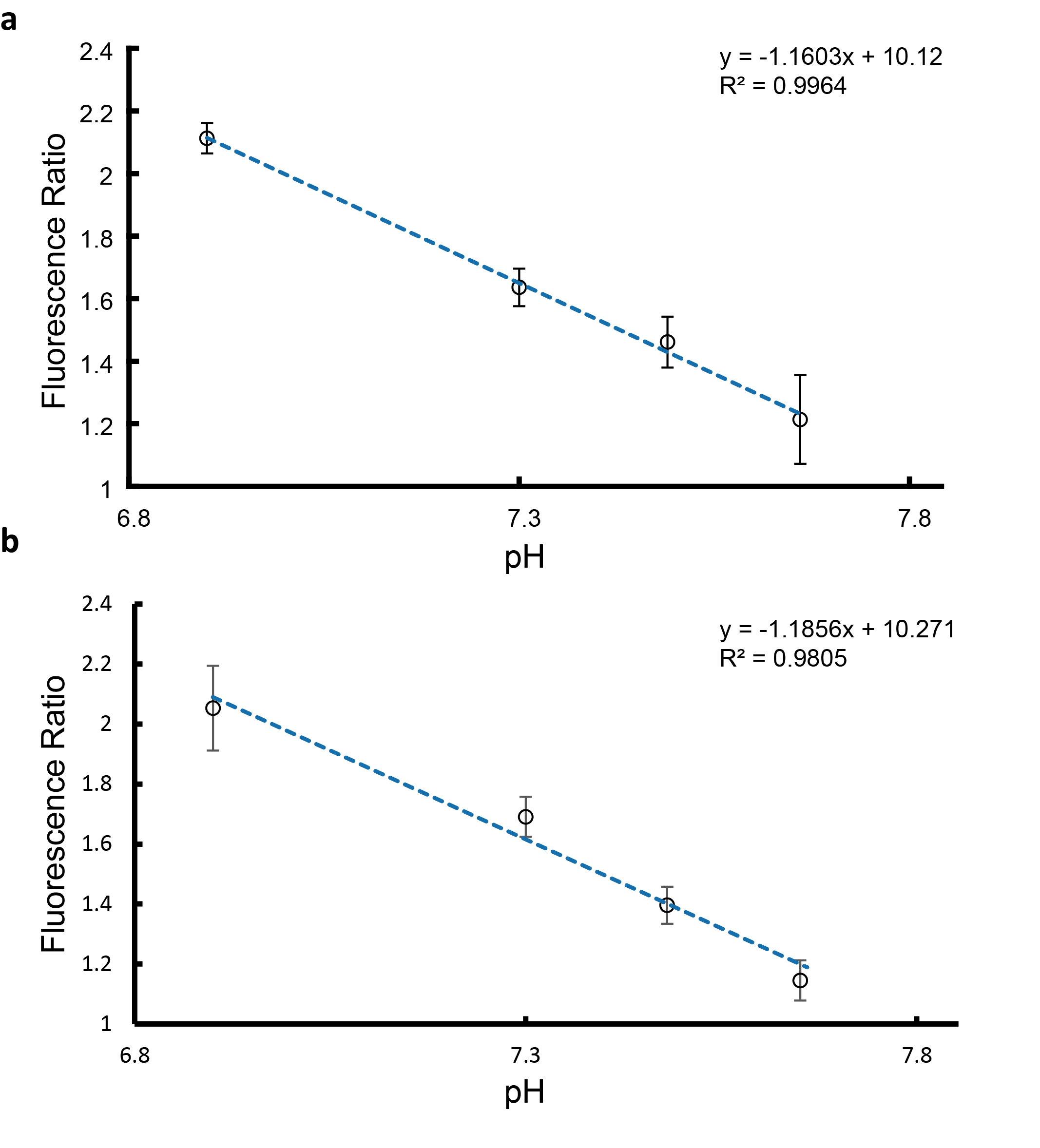


**Figure S1.** **(a)** Calibration curve quantifying the ratiometric fluorescent intensity of the SNARF-1 conjugated microparticles in Matrigel as a function of pH. **(b)** Calibration curve quantifying the ratiometric fluorescent intensity of the microparticles on top of a Caco-2 monolayer as a function of pH.

Taken together, these results suggest that although the absolute value of the luminal pH of a Caco2 cyst may be slightly lower than 7.85, it is always more alkaline than its surrounding.

References

1. Ashley, G. W., Henise, J., Reid, R. & Santi, D. V. Hydrogel drug delivery system with predictable and tunable drug release and degradation rates. *Proc. Natl. Acad. Sci. U. S. A.* **110,** 2318–23 (2013).

2. Schneider, E. L., Henise, J., Reid, R., Ashley, G. W. & Santi, D. V. Hydrogel drug delivery system using self-cleaving covalent linkers for once-a-week administration of exenatide. *Bioconjug. Chem.* acs.bioconjchem.5b00690 (2016). doi:10.1021/acs.bioconjchem.5b00690

3. Santi, D. V., Schneider, E. L., Reid, R., Robinson, L. & Ashley, G. W. Predictable and tunable half-life extension of therapeutic agents by controlled chemical release from macromolecular conjugates. *Proc. Natl. Acad. Sci.* **109,** 6211–6216 (2012).

4. Aon, J. C. & Cortassa, S. Fluorescent measurement of the intracellular pH during sporulation of *Saccharomyces cerevisiae*. *FEMS Microbiol. Lett.* **153,** 17–23 (1997).

5. Blank, P. S. *et al.* Cytosolic pH measurements in single cardiac myocytes using carboxy-seminaphthorhodafluor-1. *Am. J. Physiol.* **263,** H276–84 (1992).

6. Vecer, J., Holoubek, A. & Sigler, K. Fluorescence behavior of the pH-sensitive probe carboxy SNARF-1 in suspension of liposomes. *Photochem. Photobiol.* **74,** 8–13 (2001).

7. Hunter, R. C. & Beveridge, T. J. Application of a pH-Sensitive Fluoroprobe ( C-SNARF-4 ) for pH Microenvironment Analysis in Pseudomonas aeruginosa Biofilms Application of a pH-Sensitive Fluoroprobe ( C-SNARF-4 ) for pH Microenvironment Analysis in Pseudomonas aeruginosa Biofilms. **71,** 2501–2510 (2005).

8. Seksek, O., Henry-Toulmé, N., Sureau, F. & Bolard, J. SNARF-1 as an intracellular pH indicator in laser microspectrofluorometry: A critical assessment. *Anal. Biochem.* **193,** 49–54 (1991).
